# Osteochondral tissue engineering: scaffolds, stem cells and applications

**DOI:** 10.1111/j.1582-4934.2012.01571.x

**Published:** 2012-09-26

**Authors:** Patcharakamon Nooeaid, Vehid Salih, Justus P Beier, Aldo R Boccaccini

**Affiliations:** a Department of Materials Science and Engineering Institute of Biomaterials, Friedrich-Alexander-University of Erlangen-NürnbergErlangen, Germany; b Eastman Dental Institute, UCLLondon, United Kingdom; c Department of Plastic and Hand Surgery, University Hospital of Erlangen Friedrich-Alexander-University of Erlangen-NürnbergErlangen, Germany

**Keywords:** osteochondral tissue engineering, bilayered scaffolds, scaffold designs, scaffold fabrication, composites, clinical relevance

## Abstract

Osteochondral tissue engineering has shown an increasing development to provide suitable strategies for the regeneration of damaged cartilage and underlying subchondral bone tissue. For reasons of the limitation in the capacity of articular cartilage to self-repair, it is essential to develop approaches based on suitable scaffolds made of appropriate engineered biomaterials. The combination of biodegradable polymers and bioactive ceramics in a variety of composite structures is promising in this area, whereby the fabrication methods, associated cells and signalling factors determine the success of the strategies. The objective of this review is to present and discuss approaches being proposed in osteochondral tissue engineering, which are focused on the application of various materials forming bilayered composite scaffolds, including polymers and ceramics, discussing the variety of scaffold designs and fabrication methods being developed. Additionally, cell sources and biological protein incorporation methods are discussed, addressing their interaction with scaffolds and highlighting the potential for creating a new generation of bilayered composite scaffolds that can mimic the native interfacial tissue properties, and are able to adapt to the biological environment.

- Introduction
- Scaffolds for osteochondral tissue engineering
  – Requirement of scaffolds for osteochondral tissue engineering
  – Materials for osteochondral scaffolds
  – Cells and bioactive molecules for osteochondral tissue engineering
- Scaffold designs for osteochondral tissue engineering
  – The bilayered scaffold approach
  – Scaffolds for individual bone and cartilage tissue regeneration combined at the time of implantation
  – Scaffolds for bone component and scaffold-free approach for cartilage component
  – Single and homogeneous scaffolds
  – Single but heterogeneous scaffolds
- Discussion
- Conclusion

## Introduction

**Fig 1:**
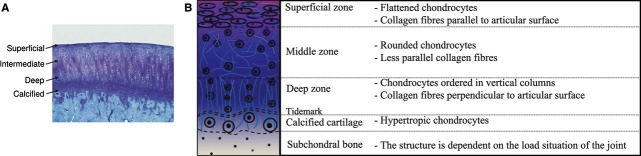
Schematic diagram of osteochondral tissue: (A) histological appearance of the osteochondral transition in rabbit articular cartilage (toluidine blue staining, ×10 magnification) (Image courtesy of Dr. Cathal O'Flatharta (Regenerative Medicine Institute, Galway, Ireland)); and (B) a diagram depicting the cartilage–bone interface.

Recent studies in the field of engineering of tissue interfaces [1–3] are leading to promising approaches for the regeneration of a variety of interface tissue defects, especially the cartilage–bone (osteochondral) interface [2]. Osteochondral defects affect both the articular cartilage and the underlying subchondral bone. Cartilage can be distinguished into four distinct zones: superficial, middle, deep and calcified cartilage zones, as depicted in Figure 1. Each zone is defined by a particular composition and organization of cells and extracellular matrix (ECM) molecules, with different proportions of ECM components significantly influencing the mechanical properties of each area [4, 5]. For example, the compressive modulus of superficial, middle and deep zones is 0.079, 2.1 and 320 MPa, respectively, indicating the notable differences in stiffness of this tissue. Bone is a complex tissue consisting of water, collagen type I and hydroxyapatite crystals, with the two latter components providing the tissue's stiffness and compressive strength [4, 6]. The compressive modulus of subchondral bone (5.7 GPa) is higher than that of cartilage. The different compositions and mechanical properties of bone and cartilage indicate the complexity of this tissue interface, making it challenging for the design and fabrication of tissue engineering scaffolds [4, 7].

Osteochondral composite scaffolds are being considered for restoration of the biological and mechanical functionality of the bone–cartilage interface [2, 4, 8]. Specific biomaterial-based strategies are being proposed, including (I) different scaffolds for the bone and cartilage sides combined at the time of implantation, (II) a scaffold for the bone component, but a scaffold-free approach for the cartilage side, (III) a single homogeneous scaffold for both components and (IV) a single but heterogeneous composite scaffold [2, 9]. Moreover, interface tissue engineering requires biocompatible and biodegradable scaffolds with highly porous microstructures for cell attachment, proliferation and stimulation of cell growth. Several biomaterials are being investigated for interface tissue engineering, including natural materials such as proteins or carbohydrate-based polymers, including collagen, hyaluronan and chitosan, and synthetic materials such as bioactive ceramics and a wide range of synthetic polymers [4, 5, 10–19]. Natural materials may enhance biological interaction with host tissues. However, the clinical applications of such natural materials are still difficult to realize with current technologies because of their relative mechanical inferiority and instability compared with native cartilage [15]. In contrast, synthetic materials, whilst lacking the intrinsic biocompatibility of natural materials, have important advantages, including some control of chemical, mechanical and structural properties of the scaffold and the possibility of satisfying the increasing clinical demand. Within the group of synthetic materials, bioactive ceramics such as hydroxyapatite (HA), calcium phosphates (CaP) and silicate bioactive glasses are being widely used in bone tissue engineering because of their excellent osteoconductive/osteoinductive capabilities [20–24]. However, synthetic biodegradable polymers, such as poly(caprolactone) (PCL) and, in particular, poly(α-hydroxy esters): poly(lactic acid) (PLA), poly(glycol alcohol) (PGA), and the copolymer of poly(lactic acid) and poly(glycol alcohol) (PLGA), are widely used in cartilage tissue engineering [2, 9].

Osteochondral tissue engineering requires a unique composition and organization of the scaffold, specific biological properties and mechanical requirements. This complexity has led to the development of bilayered scaffolds, the potential candidates for osteochondral tissue engineering, which should ideally promote individual growth of both cartilage and bone layers within a single integrated implant. Recent studies have incorporated homogeneous and heterogeneous bilayered biomaterial scaffolds within *in vitro* and *in vivo* environments, which will be discussed in the following sections. This review article will thus analyse osteochondral tissue engineering scaffolds, focusing on bilayered composite scaffolds, concerning materials, scaffold designs and fabrication methods. A discussion is provided on the relative advantages and disadvantages of the different concepts proposed highlighting promising avenues for further research.

## Scaffolds for osteochondral tissue engineering

### Requirement of scaffolds for osteochondral tissue engineering

It is generally accepted that scaffolds in tissue engineering operate as an artificial, and sometimes, temporary ECM, mimicking the structure and functionality of the native ECM, to physically guide or chemically inform cell response and thus promote tissue growth [9]. Osteochondral tissue engineering involves the combination of cartilage and subchondral bone, which have significant differences in biological structure, composition and mechanical properties. Additionally, cartilage tissue shows limitation in self-regeneration because the tissue is avascular and not innervated [25]. Generation of tissue-engineered osteochondral graft requires living cells and substitutes for the ECM in both cartilage and subchondral bone [26]. The tissue-engineered osteochondral scaffold should integrate with host tissue and maintain cell survival and phenotype during *in vivo* implantation. Mesenchymal stem cells (MSCs) have been suggested for osteochondral tissue engineering [27–29]. The correct selection of biomaterials, scaffold design and fabrication methods are crucial for the successful development of suitable scaffolds in an attempt to cope with the requirements of both cartilage and subchondral bone, and also to eliminate the problems of other approaches that include inappropriate donor tissue, immune rejection and pathogen transfer. The function of articular cartilage depends partly on the mechanical support of subchondral bone. An added complexity of scaffolds for osteochondral tissue engineering is that the subchondral matrix should have structure mimicking cancellous bone with suitable mechanical strength to withstand compressive loads and have ability to bond to the softer material used to regenerate the articular cartilage [26]. As in all tissue engineering strategies, it is necessary that the osteochondral scaffolds are highly porous with an interconnected 3-dimensional pore network for cell growth and transport of nutrients and removal of subsequent metabolic waste. The scaffold's architecture defines the ultimate shape of the newly formed cartilage and bone [12]. Scaffolds fabricated from biocompatible materials should not elicit immunological or foreign body reactions. Furthermore, scaffolds have to be chosen to be degraded and be resorbed at a controlled rate at the same time as cells seeded into the 3D construct attach, spread and proliferate, *e.g*. forming new tissue [12]. Osteochondral scaffolds should have suitable surface chemistry and topography for cell attachment, proliferation and differentiation, and the mechanical properties of osteochondral scaffolds must be tailored to match those of the host tissues at the site of implantation.

### Materials for osteochondral scaffolds

Current osteochondral-based research concepts are centred on the use of four major groups of materials: natural and synthetic polymers, metallic materials, inorganic materials (ceramics, glasses) and composites of these.

#### Natural polymers

Natural polymers, such as collagen, glycosaminoglycan, chitosan, starch, hyaluronic acid, alginate and bacterial-sourced poly(hydroxyalkanoates), are comparatively weaker and softer materials than ceramics, but offer the advantage of flexibility to adapt their shape to required forms through a variety of moulding and casting techniques [4]. Moreover, natural polymers usually contain specific molecular domains that can support and guide cells at various stages of their development [10] and thus can enhance biological interaction of the scaffold with the host tissue. Petrenko *et al*. [29] investigated Ca-alginate scaffolds with covalently attached gelatin to improve cell adhesion and proliferation. The scaffolds not only improved adhesion and proliferation of MSCs but were also able to affect MSCs to differentiate into osteogenic and chondrogenic cell lineages. The results indicated that coupling of gelatin in alginate-based scaffolds could be useful for bone and cartilage tissue engineering. Wang *et al*. [30] studied the growth of chondrocytes-seeded 3D porous poly(3-hydroxybutyrate-co-3-hydroxyhexanoate) (PHBHHx) scaffolds for cartilage repair. PHBHHx is a promising biomaterial because of its biocompatibility, resorbability and elastomeric properties. An *in vitro* study of chondrocyte-seeded PHBHHx scaffolds for 30 days showed accumulation of ECM components including collagen type II. After 16 weeks of transplantation in the knee of rabbit, cartilaginous tissue filled the defects and the constructs showed good subchondral bone connection and surrounding cartilage infusion. It was concluded that PHBHHx is an attractive material for cartilage tissue engineering. In addition, chitosan is widely studied for cartilage scaffolds [31–33] due to its structure is similar to glycosaminoglycans (GAGs) that found in ECM of articular cartilage, which influence the modulation of morphology, differentiation and function of chondrocytes. Moreover, collagen-based materials [34, 35] are considered to be a favorable biomaterial for both cartilage and bone scaffolds due to collagen is the major matrix component in ECM; collagen type II in articular cartilage and collagen type I in bone. However, immunogenic, scale-up and purification issues relevant to the clinical use of natural polymers represent important challenges [9].

#### Synthetic polymers

Biodegradable synthetic polymers include polyesters such as PLA, PGA, and PLGA, PCL, poly(propylene fumarate), poly(dioxanone), polyorthoesters, polycarbonates, polyanhydride and polyphosphazenes. They offer a wide range of chemistries and processing options and they may be obtained with controlled distribution of molecular weights [10]. The laboratory fabrication of synthetic polymers can be scaled up to industrial-scale manufacturing processing, which is a requirement to meet potential clinical demands [9]. In general, synthetic polymers have limitations in bioactivity because of their hydrophobic surface. Shafiee *et al*. [36] investigated the potential of PVA/PCL nanofibrous scaffolds for cartilage tissue engineering. PVA was selected to be electrospun with PCL to improve hydrophilicity and to support cell attachment. In an *in vitro* study, PVA/PCL scaffolds showed the proliferation and chondrogenic differentiation of MSCs. Moreover, an *in vivo* study in rabbit model, MSC-loaded PVA/PCL scaffolds supported the regeneration of cartilage and it was observed that cartilage tissue filled full-thickness defects. Additionally, development of biodegradable synthetic polymers/bioceramic incorporated composite-based scaffolds enhanced their hydrophilicity, bioactivity and elastic modulus, as shown in the studies of Huang *et al*. [37], Blaker *et al*. [38] and Ngiam *et al*. [39].

#### Metallic materials

Metals have extended applications in orthopaedic implants such as titanium, titanium alloys, stainless steels and Cobalt-base alloys [40]. In osteochondral tissue engineering, metallic biomaterials have been used as the subchondral bone part in bilayered scaffolds, which have capability of withstanding compressive loads and ability to bond to the softer material used as scaffold for the cartilage part. For example, in the study of Bal *et al*. [26], porous tantalum metal was bonded with PEG hydrogel loaded with MSCs forming a bilayered osteochondral construct. After implantation in defects of rabbit knees for 12 weeks, porous tantalum induced growth of subchondral bone and showed integration to adjacent host bone. Regeneration of hyaline cartilage-like tissue and expression of collagen type II in the articular cartilage were observed. In addition, the MSC-seeded tantalum constructs showed both osteoconductivity and osteoinductivity. Stem cell-coated titanium implants have been also investigated to provide a basis for the treatment of large osteochondral defects [26]. However, disadvantages of metallic materials in tissue engineering applications are their lack of degradation over time and the possibility of release of wear particles or corrosion products [40].

#### Inorganic materials

Ceramics, such as HA or other calcium phosphates, such as TCP and bioactive glasses, such as Bioglass®, are known to promote biomineralization and they are widely used in bone tissue engineering [2, 12, 41]. When implanted, these materials promote the formation of a bone-like apatite layer on their surface, leading to bone-bonding behaviour and assuring enhanced fixation of the scaffold to the host tissue. These materials have excellent osteoconductivity and osteoinductivity. For instance, Mastrogiacomo *et al*. [42] suggested that 100% HA porous scaffolds have good osteoconductive properties resulting in enhanced bone formation. After implanting MSC-seeded scaffolds in a murine model, it was observed that bone was formed and the amount of bone formed increased with culture time. Additionally, bone formation was enhanced on scaffolds with higher porosity and exhibited a higher degree of interconnection among pores. This study concluded that HA is a promising biomaterial for bone reconstruction [42]. Ideal scaffolds for bone repair require an internal architecture similar to that of natural bone to promote cell proliferation and cell ingrowth into the structure. Leukers *et al*. [22] used a 3D printing technique to fabricate scaffolds based on HA with complex internal structure. From the *in vitro* study of cell-seeded scaffolds, it was confirmed that cells could proliferate deep into the structure of the HA scaffolds without clogging. Xynos *et al*. [43] used 45S5 Bioglass®-based scaffolds to enhance bone formation by the effect of ion dissolution products (Hoppe *et al*. [44]). It was found that these scaffolds induced osteoblast proliferation and differentiation. These findings confirmed the applications of Bioglass® in bone tissue engineering where the Bioglass® substrate is proposed as a template for the formation of bioengineered bone tissue. In this context, Bioglass®-based scaffolds have been fabricated by the foam replication method, exhibiting pore structure resembling cancellous bone [45]. In addition, an alternative bioactive glass, BG 13-93 was studied by Jayabalan *et al*. [46] to investigate its effect on chondrocytes behaviour and its use in cartilage regeneration. A porous BG 13-93-based scaffold was used as a subchondral substrate and chondrocyte-loaded agarose was used as cartilaginous substrate in a bilayered osteochondral construct. After culturing for 6 weeks, the results did not follow the hypothesis that BG 13-93 substrate would increase production of GAG and collagen in the cartilage layer. In contrast, cell-seeded agarose exposed to BG 13–93 showed improvement of GAG and collagen production in cartilage layer. Therefore, BG 1393 was considered to have potential as a culture medium supplement for cartilage regeneration, but it might be not suitable for application as scaffold in osteochondral constructs.

Moreover, bioceramic-based scaffolds exhibit suitable stiffness, but they have low structural integrity being brittle and unsuitable for application under mechanical stresses. The degradation behaviour of inorganic scaffolds can be controlled by changes in the porous structures, which can be tailored in terms of their degradation kinetics appropriate for bone tissue engineering. It is also well known that increasing porosity impairs further the mechanical properties of bioceramic scaffolds. This problem can be solved by modifying porous inorganic scaffold with infiltration or coating by biodegradable polymers. As shown in a study by Miao *et al*. [47], porous HA/TCP scaffolds exhibited improved mechanical properties by coating with PLGA. The coating with PLGA could improve the compressive strength by about 10 fold compared with that of uncoated scaffolds, whereas the porosity slightly decreased by 2%. Similar results were observed by Chen *et al*. [45] in a study of PDLLA-coated 45S5 Bioglass®-based scaffolds. The compressive strength was slightly improved and the work of fracture (related to the energy required to induce fracture in the material) was significantly enhanced by the PDLLA coating. Moreover, the bioactivity of Bioglass®-based scaffolds was maintained after application of PDLLA coatings. Several studies on polymer-coated inorganic scaffolds for bone tissue engineering, including HA, bioactive glass, Al_2_O_3_, TiO_2_, ZrO_2_ and CaP, have been reviewed by Yunos *et al*. [48], showing that these scaffolds achieve suitable mechanical properties for this application.

In osteochondral tissue engineering, biomaterials which can fulfil the ideal requirements are still being sought. In this context, individual bioceramic-based scaffolds and pure polymer-based scaffolds have limitations for the repair of osteochondral defects. Therefore, new approaches based on composite materials for osteochondral tissue engineering are being proposed as described below.

### Cells and bioactive molecules for osteochondral tissue engineering

In osteochondral repair, chondrogenic and osteoblastic cell lines have been widely used for clinical applications; however, adult and embryonic stem cells have recently received great attention [49, 50]. Stem cells are an alternative resource to overcome the limited supply of primary cells [51]. In addition, specific signalling molecules, such as growth factors (TGF-β family, insulin-like growth factors, bone morphogenetic proteins and fibroblast growth factors), are extensively employed to facilitate tissue growth [51]. They bind to cell surface receptors and activate intracellular signalling pathways, which affect cell proliferation, differentiation and ECM synthesis during tissue regeneration [51]. Among the growth factors, the TGF-β family could enhance the synthesis of the cartilage ECM in chondrocytes and induce chondrogenesis in stem cells. Chondrocytes (articular, auricular, costa, nasoseptal) and osteoblasts are an obvious choice because they are found in native cartilage and bone, respectively, and are generally relatively simple to access surgically. Sherwood *et al*. [52] produced bilayered scaffolds based on PLGA/TCP composite for the bone and PLGA/PLA for the cartilage area, respectively, which were seeded with ovine articular chondrocytes and cultured for 6 weeks. The results showed the formation of cartilage, but *in vivo* investigations were not performed to verify the performance of these scaffolds. The use of chondrocytes in such 2-dimensional *in vitro* systems may have limitations in cell re-differentiation, which leads to the down-regulation of cartilage specific genes. In this context, the reduced capacity of expanded chondrocytes to re-differentiate and produce cartilage-specific ECM may impair the goal of generating functional cartilage tissue [2]. This problem can be overcome by expanding chondrocytes in the presence of specific growth factors. For instance, Allan *et al*. [53] used bovine chondrocytes with porous CPP scaffolds and cultured them in the presence of sodium β-glycerophosphate (Na-β-GP) for 8 weeks to generate a calcified cartilage zone. After culturing, two layers of cartilage tissue were observed corresponding to the hyaline-like and the calcified cartilage zones. When compared with the control condition, little or no mineralization occurred without the addition of Na-β-GP. The presence of the differentiation factor during expansion of chondrocytes showed not only to increase cell proliferation but also to enhance the cell capacity to re-differentiate. Moreover, another approach for osteochondral tissue engineering is to reconstruct the functional engineered cartilage–bone interface by co-culturing chondrocytes and osteoblasts into bilayered scaffolds, for growth and differentiation of both cell types [54]. Cao *et al*. [55] designed and fabricated 3D porous PCL scaffolds by fused deposition modelling (FDM). Osteoblasts were seeded and precultured into one half of the scaffold, and later chondrocytes were seeded into the other half. After that, the cell-seeded scaffolds were cultured in a co-culture medium, both cell types proliferated, migrated and integrated at the interface. Moreover, osteoblasts and chondrocytes produced different ECM in each own compartment. In this approach, the important aspect is the interactions between both cell types and the scaffold during co-culture, and the assessment of material-dependent effects on the formation of the functional bone–cartilage interface. Moreover, it is critical to control both osteogenic and chondrogenic phenotypes during co-culture to maintain each tissue zone similar to native tissue. Stem cells (bone marrow-derived, adipose-derived, muscle-derived, synovium-derived, periosteum-derived, embryonic), are also significantly interesting for osteochondral tissue engineering. In 1998 (Johnstone *et al*. [56]), MSCs were found to undergo chondrogenesis when cultured in the presence of TGF-β1. The addition of TGF-β1 has been shown to stimulate chondrogenesis; however, the degree of chondrogenesis also depends on the scaffold. Gao *et al*. [57] used a bilayered composite to mimic the natural tissue geometry, which was composed of MSCs-seeded injectable calcium phosphate (ICP) and osteogenic supplement for bone regeneration and a hyaluronan layer seeded with MSCs and TGF-β1 for cartilage regeneration. After 12 weeks of implantation, the composites displayed collagen type I in neotissue in both components and collagen type II in fibrocartilage. This composite osteochondral graft showed that its construction of different biomaterials and bioactive factors can support either chondrogenic or osteogenic differentiation of MSCs. Alhadlaq *et al*. [58] prepared osteochondral scaffolds with PEG hydrogel seeded with rat MPCs. After 4 weeks of implantation in mice, most differentiated chondrogenic and osteogenic cells had synthesized both corresponding cartilaginous and bone-like ECM. Overall, the ideal cell source must be easily isolated, capable of expansion, and cells should be cultured to express and synthesize at least aggrecan and collagen type II on the cartilage region, and provide mineralization on the bone region. Moreover, growth factors have been used to induce cell proliferation and extracellular molecules synthesis in culture [14]. In a study by Huang *et al*. [59], an implant comprising PCL scaffold, MSCs and TGF-β1 growth factor-loaded fibrin glue was investigated to assess the induction of cartilage formation when implanted in a lapine model. It was found that at 4 weeks after implantation, the scaffolds were richly populated with chondrocytes and immature bone was identified at 6 weeks after implantation because of the presence of growth factors that acted as recruitment agents to attach cells to the site. Traditionally, TGF-β1 has been regarded a cartilage-inducing factor as well as a bone-inducing factor. Abrahamsson [60] prepared 3D woven scaffolds based on PCL seeded with human MSCs and cultured in the presence of human TGF-β3 growth factor to analyse chondrogenesis and mineralization on osteochondral scaffolds. After culture for 21 days, formation of cartilaginous tissue was observed. Mineralization was observed in the newly formed ECM at the interface with the underlying scaffold by day 45. The construct after implantation for 45 days also showed mechanical properties similar to those of native articular cartilage. These findings merit consideration when developing grafts for osteochondral defect repair. Overall, MSCs are an attractive cell source for osteochondral tissue engineering [27, 61]. Especially, these cells provide higher proliferative capacity and have ability to differentiate into osteoblasts or chondrocytes in different simulative environments, [62] although they yield lower initial cell numbers compared with primary cells [6, 10, 11]. The primary cells (chondrocytes and osteoblasts) might have limitation in their use because of more risk of causing phenotypic changes and age-dependent proliferative capacity [6]. Nevertheless, chondrocyte hypertrophy in neocartilage (*e.g*. collagen type X) could be expressed by MSCs undergoing chondrogenesis without any bioactive factors, which results in apoptosis, vascular invasion and ossification [28]. Thus, the interaction between specific cells and material substrates, and the effect of bioactive factors or supplements must be carefully investigated in this area.

## Scaffold designs for osteochondral tissue engineering

### The bilayered scaffold approach

The scaffold design is paramount for the success of osteochondral tissue engineering, being necessary to consider the scaffold microstructure, surface topography, porosity, pore geometry and orientation, biodegradability and mechanical properties of scaffolds. These scaffold characteristics dictate the ability of the scaffold to resist mechanical loading and influence the associated tissue regeneration. As a result of bone and cartilage having uniquely different compositions, tissue growth mechanisms and metabolic requirements, respectively, bilayered composite structures have been developed to exploit and combine the advantages of various individual materials. Bilayered scaffolds allow for creation of optimized tissue-specific biological environments in each layer *via* variation of the local mechanical, structural and chemical properties. In addition, bilayered scaffolds can be designed to mimic the native ECM for each tissue type independently, which may be more convenient than intending the fabrication of monolithic constructs with different functional requirements of both bone and cartilage in a single structure [9]. Bilayered scaffolds have been classified into four types [2, 9], as mentioned above. The summary of various approaches being followed in the design of bilayered composite scaffolds, including materials and fabrication methods, is shown in Table 1.

**Table 1:** Summary of bilayered scaffolds developed for osteochondral tissue engineering

Scaffold strategy	Materials		Fabrication	Model system for osteochondral defect	*In vitro/in vivo*	References

	Cartilage part	Subchondral bone part				
Different scaffolds for individual bone and cartilage tissue combined at the same time of implantation	PGA mesh	PLGA/PEG composite	Sutured	Articular chondrocytes seeded onto PGA meshes and periosteal cells seeded onto PLGA/PEG scaffold	*In vitro*	Shaefer *et al*. [64]
	PGA mesh	Collagraft matrix	Sutured	Chondrocytes seeded on PGA meshes	*In vivo* in rabbit	Shaefer *et al*. [70]
	Hyaluronic acid	CaP	Press fitting	Chondrogenic MPCs seeded on hyaluronic acid sponge and osteogenic MPCs seeded on CaP scaffold. Two parts were sealed with fibrin glue.	*In vivo*	Gao *et al*. [57]
	PCL	PCL/TCP composite	Press fitting	Bone marrow mesenchymal cells seeded into both parts individually	*In vitro*	Shao *et al*. [65]
	Fibrin glue	PCL	Fused deposition modelling (FDM)	Bone marrow mesenchymal cells seeded into both parts individually	*In vitro*	Shao *et al*. [65]
	Collagen-glycosaminoglycan	Collagen-glycosaminoglycan/CaP	Freeze drying technique	–	*In vitro*	Harley *et al*. [141]
	Collagen	Fibrin gel	Press fitting	Human chondrocytes seeded on collagen	*In vitro*	Scotti *et al*. [68]
	PLGA microspheres	PLGA microspheres	Press fitting	Transforming growth factor (TGF-β1) loaded microspheres for cartilage and bone morphogenetic protein (BMG-2) loaded microspheres for bone	*In vivo* in rabbit	Dormer *et al*. [62]
Scaffolds for bone component but scaffold-free for cartilage component	Chitosan/gelatin (CG)	Hydroxyapatite/chitosan/gelatin (HCG)	Freeze drying technique/fibrin glue	MSCs seeded in pTGF-β1 loaded CG scaffold and pBMP-2 loaded HCG scaffold	*In vivo* in rabbit	Chen *et al*. [69]
	–	PLLA Collagen/HA PDLLA	Thermally induced solid-liquid phase separation	Chondrogenic cells seeded	*In vivo* in sheep	Wang *et al*. [72]
	–	PLA	–	Human MPC seeded onto PLA and pre-cultured in chondrogenic medium.	*In vitro*	Tuli *et al*. [11]
	–	β-TCP	Sintering process	Osteogenic cells seeded onto opposite side of PLA scaffold	*In vitro*	Guo *et al*. [73]
	-	CPP	Sintering process	Articular chondrocytes seeded onto β-TCP scaffold	*In vitro*	Kandel *et al*. [74]
	–	Bone matrix gelatin (BMG)	Sintering process	Chondrocytes seeded onto porous CPP scaffold	*In vitro*	Li *et al*. [76]
	–	CPP	Sintering process	Rabbit chondrocytes seeded	*In vivo* in sheep	Allan *et al*. [53]
	–	HA	Sintering process	Chondrocytes seeded onto porous CPP scaffold	*In vivo* in mice	Kitahara *et al*. [77]
	–	Woven PCL fibres	–	Alginate-recovered chondrocytes (ARC) from rabbit seeded on HA scaffold	*In vitro*	Abrahamsson *et al*. [60]
Single and homogeneous scaffolds	PEG hydrogel	PEG hydrogel	Photopolymerization	Chondrogenic rat MPCs embedded in one half of PEG hydrogel and osteogenic rat MPCs embedded in another half of PEG hydrogel	*In vivo* in knee of beagle	Alhadlaq *et al*. [58]
	Fibrous PE scaffold coated with HA	Fibrous PE scaffold coated with HA	–	Growth factor FGF-2 loaded in PE fibre scaffold	*In vivo* in rabbit	Fukuda *et al*. [142]
	Collagen	PLGA/collagen composite	Collagen in both layers was connected	Bone marrow derived mesenchymal stem cells cultured in scaffold	*In vivo* in rabbit	Chen *et al*. [79]
	Chitosan	HA	Press fitting	Bone marrow derived mesenchymal stem cells cultured in scaffold	*In vitro*	Oliveira *et al*. [80]
	Fibrous collagen (I/III) matrix	TCP	–	Additional growth factor mixture (GFM) loaded in scaffold	*In vivo* in knee of beagle	Gotterbarm *et al*. [81]
	CPP/gelatine	Porous CPP	–	Rat's joint chondrocytes seeded into scaffold	*In vivo* in knee of beagle	Lien *et al*. [82]
	pCol-HA/ChS	pCol-HA/ChS	–	Chondrogenic MSCs seeded into scaffold	*In vivo* in mice	Ohyabu *et al*. [2000]
	Porous collagen	Dense collagen	Freeze drying technique	Chondrocytes seeded into collagen matrix scaffold	*In vitro*	Frenkel *et al*. [18]
Single but heterogeneous scaffolds	Oligo(poly(ethylene glycol) fumarate) (OPF)/gelatin microparticles	OPE	Cross-linking	Growth factor β1 loaded microparticle on OPF/gelatin microparticle	*In vivo* in rabbit	Holland *et al*. [78]
	PLA/starch	HA	–	Growth factor β1 loaded microparticle on OPF/gelatin microparticle	*In vitro*	Ghosh *et al*. [94]
	Collagen	PLGA/Collagen	–	Bone marrow mesenchymal cells seeded into scaffold	*In vivo* in canine	Chen *et al*. [79]
	PDLLA mesh	PDLLA-coated Bioglass®-based scaffold	Electrospinning/Foam replication method	Chondrocytes seeded on PDLLA electrospun fibres	*In vitro*	Yunos *et al*. [48]
	Collagen/chitosan	Collagen/58S BG	Collagen in both layers was connected/Cross-linking	Bone marrow mesenchymal cells seeded into scaffold	*In vitro*	Bi *et al*. [91]
	PLA	HA	Solid free-form technique (SFF)	Articular chondrocytes seeded into PLA and human fibroblast BMP-7 seeded into HA	*In vivo* in mice	Schek *et al*. [93, 145]
	PLGA/PLA composite	PLGA/TCP composite	3D printing process	Articular chondrocytes seeded in PLGA/PLA region	*In vitro*	Sherwood *et al*. [52]
	PCL	PCL	Moulding	Human rib chondrocytes seeded in one half of PCL scaffold and MPCs cultured in another half of PCL scaffold	*In vitro*	Cao *et al*. [55]
	Porous PLGA	Porous PLGA/TCP composite	Fused deposition modelling (FDM)	Articular chondrocytes seeded in PLGA region	*In vivo* in mice	Liao *et al*. [143]
	Agarose hydrogel	PLGA/45S5Bioglass® microsphere composite	Moulding	Chondrocytes encapsulated in Agarose hydrogel and osteoblasts were seeded in PLGA/45S5Bioglass® microsphere composite	*In vitro*	Jiang *et al*. [95]
	Elastin-like recombinamers (ELRs)/collagen fibrous mesh	Collagen	Freeze-drying/Electrospinning	Culture of human fibroblasts and epithelial cells	*In vitro*	Kinikoglu *et al*. [97]
	Collagen fibres in microspheres	Collagen fibres in microspheres	Undifferentiated MSCs-collagen microspheres as a glue	Rat mesenchymal stem cells (rMSCs)	*In vitro*	Cheng *et al*. [137]
	Ethylene vinyl acetate (EVA)	Polyamide66 (PA66)/hydroxyapatite (HA)	Co-precipitation and thermal-induced phase inversion method	MG63 cells	*In vitro*	Luo *et al*. [144]

### Scaffolds for individual bone and cartilage tissue regeneration combined at the time of implantation

**Fig 2:**
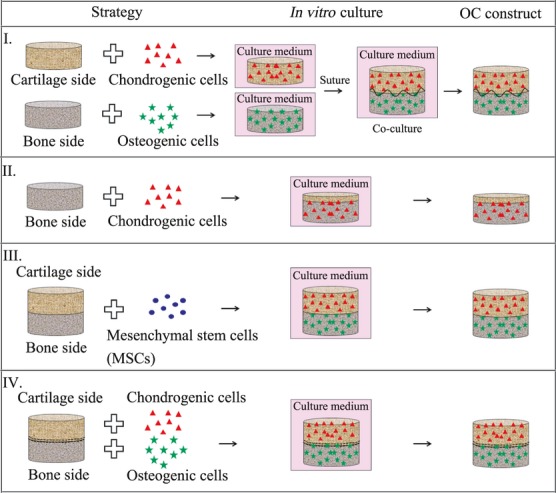
Schematic diagram of bilayered scaffolds, including (I) scaffolds for individual bone and cartilage tissue regeneration combined at the time of implantation, (II) scaffold for bone component and scaffold-free approach for cartilage component, (III) single and homogeneous scaffolds and (IV) single but heterogeneous scaffolds (Modified from Mano *et al*. [10]).

Because of the markedly different tissue properties at the interface between cartilage and subchondral bone, different scaffolds can be used individually for cartilage and bone components. If bioreactors are used, they allow the cultivation of both chondrogenic and osteogenic cells in separate environments [63]. Then, constructs for cartilage tissue and bone tissue developed separately are combined into a single composite graft by suturing or adhering two layers together, as shown in Figure 2 (I). The main disadvantage of this approach is that integration between the two layers may not be satisfactory [10]. Schaefer *et al*. [64] investigated the use of 3D cartilage/bone composites based on biodegradable polymer scaffolds combined with chondrogenic and osteogenic cells. Cartilage constructs were created by PGA meshes cultured with bovine calf articular chondrocytes. Bone constructs were created by the blend of PLGA and PEG cultured with bovine calf periosteal cells. Pairs of constructs were sutured together after 1 or 4 weeks of isolated culture, then the resulting composites were cultured for additional 4 weeks. It was found that the osteochondral composites generated by suturing were stable and did not separate upon removal of the sutures at the time of harvest. From the histological assessment, the accumulation of GAG showed a higher scattered area at 4 weeks culture time in comparison with that at 1 week culture time. In the region of bone, no evidence of mineralization was found after 1 week, whereas there was mineralization after culturing for 4 weeks. Therefore, the amount of GAG and mineralization was confirmed to increase with cultured time. After 1 week of culture of the cartilage compartment combined with 1 week of culture of the bone compartment and with additional culture of the composites for 4 weeks, good integration at the tissue interface was found. In contrast, composites obtained from the combination of 4 weeks culture of cartilage and 4 weeks culture of bone separately showed poor integration at the interface, although more continuous ECM-containing GAG was observed in the cartilage region of both composites after additional 4 weeks of combined culture. This study concluded that newly produced cartilage and bone tissues affected integration at the cartilage–bone interface. The combination of immature cartilage-like construct (after 1 week in isolated culture) and mature bone-like construct (after 4 weeks in isolated culture) was seen to be effective to form a composite construct and to promote integration at the interface. Gao *et al*. [57] demonstrated the potential of using an ICP for the bone layer and a hyaluronan (HyA) sponge seeded with MPCs for the cartilage layer. After 12 weeks of implantation in a lapine model, the osteochondral defect was filled almost 100% with repair tissue and the HyA part was resorbed by 10 weeks. Zonal arrangement, including superficial, chondroid tissue, and interface layers, appeared to take place in the neo-cartilaginous tissue [57]. Significantly, the two-phase composite construct showed great integration at the interface between HyA and ICP components, ascribed to the local mechanical stress. The compressive load of the joint was applied and transmitted through the HyA sponge, ICP component and the bottom of the defect. The counteracting load was generated upwards and laterally, and induced the infiltration of ICP into the pores of the HyA sponge, leading to interface integration. Moreover, the laterally counteracting load was expanded laterally by the HyA sponge, causing a contact between the sponge and the surrounding native cartilage. Shao *et al*. [65] attempted to evaluate the repair potential in osteochondral defects (high load-bearing sites) by using hybrid scaffolds with MSCs in a lapine model. The scaffolds comprised PCL for the cartilage component and TCP-reinforced PCL for the bone component. The scaffolds were seeded with MSCs in each part and placed in osteochondral defects of lapine models by press-fit implantation. Repair tissues were evaluated at 12 and 24 weeks after implantation [65]. Compared with the control group (without cells), the PCL/PCL-TCP scaffolds showed superior repair ability in both bone and cartilage parts, indicating that the hybrid scaffolds provided sufficient support to new osteochondral tissue formation. From a period of 12–24 weeks, bone generation led to the firm integration to host tissue. After 24 weeks of implantation, subchondral bone filled the scaffold, which showed good integration with the host bone. Moreover, cartilage tissue exhibited GAG and collagen type II deposition. However, the cell arrangement in new cartilage tissue lacked zonal organization. The Young's modulus of the neotissue–polymer matrix construct at 24 weeks after implantation (∼0.76 MPa) approached that of normal cartilage of mature rabbits (∼0.81 MPa) [65]. The authors stated that this phenomenon could have been caused by the slow degradation of PCL-based hybrid scaffolds, which might leave remnants in the repair space over time and these remnants could help maintain sufficient mechanical support for subchondral bone and neocartilage. However, the possible changes in the scaffold and repair tissues over longer times of implantation were not shown in the study [65]. Neocartilage that deteriorates with time may happen, as shown in the study of Chu *et al*. [66]. Neocartilage in the region of repair tissue in rabbit decreased from 95% at 12 weeks to only 29% at 1-year follow-up [66, 67]. Scotti *et al*. [68] generated osteochondral composites including collagen-containing human chondrocytes for the cartilage part and fibrin gel for the bone part. It was shown that the separate cell pre-culture before generation of the composite allowed more efficient cartilaginous matrix accumulation than without pre-culture. Moreover, good biological bonding of the chondral scaffold with the bony scaffold by the cell-laid ECM occurred, indicating a suitable mechanical integrity at the interface and the possibility of effective surgical handling. Chen *et al*. [69] formulated a bilayered scaffold for simultaneous regeneration of cartilage and bone using gene delivery system to induce the growth of MSCs. Plasmid TGF-β1 activated chitosan/gelatin (CG) porous scaffold and Plasmid BMP-2 activated hydroxyapatite/chitosan/gelatin porous (HCG) scaffold were fabricated for the cartilage and bone regions, respectively. Both scaffolds were seeded with MSCs separately before integrated with fibrin glue. The interface of the bilayered scaffold showed good integration as a result of the interdigitation of the chondral phase into the osseous phase. After 2 weeks of co-culture, it was found that pTGF-β1 and pBMP-2 can induce MSCs in each layer to differentiate into chondrogenic and osteogenic-like cells. This demonstrated that the localized delivery system of DNA as tissue inductive factors in bilayered scaffolds could facilitate the differentiation of stem cells into specific cell types to develop complex tissues. An *in vivo* study in a rabbit model showed that the gene delivery system utilized in this bilayered construct simultaneously supported cartilage and bone regeneration, presenting a promising strategy for facilitating the development of osteochondral tissue [68].

In summary, scaffolds for individual bone and cartilage tissue regeneration combined at the time of implantation represent one of the scaffold designs for osteochondral repair, based on the combination of different materials and cells specified for individual cartilage and subchondral bone tissues or different biological factors to induce the differentiation of stem cells into chondrocytes and osteoblasts. Pre-culture of individual scaffolds with relevant cells is an important step. Also, the isolated culture period affects integration at the interface between cartilage and bone parts. Thus, most studies have shown that good integration at the cartilage–bone interface was achieved by biological bonding of newly formed ECM produced by cell-seeded scaffolds [68, 70]. Therefore, immature cartilage or bone-like tissue constructs should be chosen for forming composite constructs before *in vivo* implantation. Also, the cartilage–bone interface can be improved by local mechanical loading of the joint. Moreover, this load can help achieve good contact between neotissue and host tissue. However, the degeneration of new formed cartilaginous tissue has been observed with longer implantation times [66, 67, 71]. One of the reasons for this result was considered to be biomechanical incompatibility occurring at the defect site, for example, low stiffness and fast degradation rate of the materials used [68]. This problem can be overcome by appropriate biomaterials, which have biocompatibility, tailored degradation rate, and sufficient strength over a long enough period of time to withstand *in vivo* physiological forces. Finally, a limitation of this strategy could be the labour-intensive work related to *in vitro* culture of engineered cartilage for 4 or 6 weeks [70], and it may not be convenient for further clinical application.

### Scaffolds for bone component and scaffold-free approach for cartilage component

Instead of using two scaffold structures, this family of bilayered scaffolds incorporates neocartilage tissue that has been grown *in vitro* from seeded chondrocytes on the top of a subchondral support to form the secondary layer [53], as shown in Figure 2 (II). This design therefore requires a suitable engineered material to support subchondral bone and to allow the induction of neocartilage tissue. There are many approaches which focus on biomaterials for this type of scaffold that include bioceramics, such as HA, Bioglass®, calcium sulphate and CaP, natural polymers, such as collagen and chitosan, and synthetic polymers, such as PLLA, PDLLA and PGA. Wang *et al*. [72] explored various candidate biodegradable support materials onto which neocartilage was produced *in vitro*. PLLA, PDLLA and collagen-hydroxyapatite (Col-HA) were seeded with chondrocytes and cultured in a closed and static bioreactor for 15 weeks. It was found that neocartilage formed onto all types of biomaterial supports. PLLA and Col-HA scaffolds were still in disc form after 15 weeks culture, whereas the PDLLA scaffolds were not regular in shape because of the collapse of the PDLLA structure, after 11 weeks in culture. As a result of the degradation of PDLLA, the highest amount of cell death was observed, indicating that this material might not be ideal for implantation, even though after removal from the chamber, the material was stable enough to be handled by forceps. In the study of Wang *et al*. [72], the composite Col-HA showed the lowest rate of degradation and the lowest number of dead cells. Moreover, cell ingrowth was observed, while there were only a few cells observed inside PLLA scaffolds. Neocartilage in Col-HA scaffolds also displayed collagen fibrils, which were well connected between cartilage tissue and subchondral bone-like tissue at the interface. It can be concluded that under the conditions of the study [72], Col-HA composite was superior in comparison with PLLA and PDLLA in terms of cell viability, construct shape and cellular integration. However, it is yet to be determined if this material is adequate for implantation from the mechanical point of view because of the loads experienced at the joint. It was predicted that superficial binding may lead to implant delamination. Guo *et al*. [73] prepared β-TCP-based scaffolds as subchondral bone construct, which was tested in a sheep model. The β-TCP porous ceramics were proposed because of their faster resorption potential compared with HA. Chondrocytes were seeded into 3D scaffolds to create cell-scaffold constructs and to evaluate their efficacy for cartilaginous tissue generation. The characteristics of these scaffolds included homogeneous porosity of 70%, spherical pores of 450 ± 50 μm in diameter and interconnections of 150 ± 50 μm in diameter. From histological examination, chondrocyte proliferation and neocartilage colonization were found inside the pores. The defects (ovine model) were entirely filled with neocartilage tissue within 24 weeks after implantation. Furthermore, β-TCP scaffolds exhibited many desired properties, such as performance stability, good compatibility, relatively high mechanical strength, suitable macrostructure, excellent osteoconduction and biodegradation properties. However, it was recommended that long-term *in vivo* observations of the host tissue response to the scaffolds should be made to validate the approach. In addition, the stability of repaired cartilage should be further investigated. Kandel *et al*. [74] developed osteochondral constructs by using porous calcium polyphosphate (CPP) as a substrate to grow articular cartilage on the surface. The CPP scaffolds were seeded with chondrocytes and studied *in vivo* in an ovine model. After implantation, there was evidence that the implant could withstand loading up to 36 weeks and it was fixed by adjacent native cartilage and bone ingrowth in the CPP substrate. However, some implants in this study showed that the cartilaginous tissue delaminated during the 12–16 weeks of implantation period because of a low cartilage/CPP interfacial shear strength in comparison with the native osteochondral interface [74, 75]. Li *et al*. [76] prepared demineralized bone matrix gelatin (BMG)-based scaffolds for osteochondral tissue engineering, which were cultured with rabbit chondrocytes to produce neocartilage. It was found that neocartilage of about 1.3 mm thickness was produced on the BMG scaffold after 6 weeks of *in vitro* culture. Hyaline-like cartilage, intense collagen type II and abundant proteoglycan were observed, whereas collagen type I and protein expression were significantly low. Moreover, it was found that the interface between cartilage and the BMG scaffold remained a weak area, caused by the fact that BMG scaffolds do not have a structure like subchondral bone that could allow chondrocytes to grow into the porous structure and form the calcified cartilage layer [76]. Allan *et al*. [53] attempted to mimic the structural arrangement of the ECM by forming a zone of calcified cartilage, using biphasic constructs composed of cartilaginous tissue anchored to the top surface of the bone substitute that was a porous CPP with calcified interface. When cultured in the presence of Na-β-GP for 8 weeks, a two-layered cartilage tissue (calcified cartilage (interface) and hyaline-like zones) was observed. In terms of mechanical properties, the equilibrium stress increased with the presence of a calcified interface zone, as shown in Table 2. Also, the equilibrium modulus, indicating tissue stiffness, of the neocartilage with a mineralized zone was significantly greater than that of the *in vitro*-formed tissue without mineralization. In addition, the interfacial shear properties at the cartilage-CPP scaffold were enhanced as a result of the efficient integration of hyaline-like cartilage and the CPP scaffold by the calcified cartilage layer. This result demonstrated the importance of the presence of a mineralized zone in bioengineered cartilage. Kitahara *et al*. [77] studied the *in vivo* performance of HA as subchondral bone scaffold for articular cartilage repair. The HA scaffolds were seeded with alginate-recovered-chondrocytes (ARC) to generate cartilage tissue for osteochondral repair. Then, bilayered scaffolds were implanted in mice. At 8 weeks of implantation, the chondrocytes were well distributed in the interface area and expression of collagen type II was observed particularly in the local area around the chondrocytes. Moreover, histological results showed a massive area of GAG from the surface to the interior of the construct. However, one limitation of this study could be that the conditions in the knee joint cavity of mice differ from those in a human joint cavity. Therefore, further investigations are required to confirm the feasibility of the ARC-HA constructs for the repair of osteochondral defects in humans. Abrahamsson *et al*. [60] studied the effect of culture time on the properties of 3D woven PCL scaffolds seeded with human MSCs. In chondrogenic medium, cartilaginous tissue formed at 3 weeks and new formed ECM were observed at the interface after 7 weeks in culture. It was confirmed that the GAG amount depended on culture time. Furthermore, the constructs showed mechanical properties similar to those of native articular cartilage. These results thus are relevant when developing grafts for osteochondral defect repair. Nevertheless, it was apparently shown that most of the above studies used bioceramic materials as a scaffold to generate both bone and cartilage tissues. Only bioceramic-based scaffolds, however, might not be successful candidates for osteochondral repair because of their hardness and lack of flexibility which polymers have. Consequently, this mismatch of mechanical properties may lead to delamination between the bone scaffold and new cartilage. Overcoming this problem is suggested by using polymer/bioceramic composites as scaffolds.

**Table 2:** Compressive mechanical properties of cartilage, according to Allan *et al*. [53]

	*In vitro-*formed cartilage(No Na-β-GP) (*n*= 7)	*In vitro-*formed cartilage(10 mM Na-β-GP) (*n*= 7)	*In vivo* osteochondral Plugs (*n* = 6)
Equilibrium stress (kPa)	3.9 ± 0.6	10.5 ± 2.3	53.1 ± 9.1
Equilibrium modulus (kPa)	18.4 ± 7.8	131.3 ± 28.3	561.2 ± 87.8

In summary, the approach involving a scaffold for the bone component, but none for the cartilage component, can be applied to form cartilaginous tissue on subchondral bone substrate. This scaffold type can reduce the possible problems associated with degradation properties and biocompatibility of scaffold materials. Some studies have shown that bilayered scaffold-free cartilage constructs exhibit *in vitro* formation of cartilaginous-like tissue by chondrocytes seeded without the aid of biomaterial support [53, 72, 74, 77]. Possible problems of acid by-product accumulation from synthetic polymers and inflammatory reactions during *in vivo* implantation can be thus minimized. However, low interfacial shear strength at the interface between cartilage and the underlying bone scaffold is still a potentially vunerable aspect of such systems. The formation of a mineralized layer in engineered cartilage has been suggested to resolve this problem [53, 76] considering that calcified cartilage is important for the integration of soft tissue (nonmineralized hyaline-like cartilage) and hard tissue (mineralized subchondral bone), and it can distribute the mechanical load across the interface [53]. Although calcified cartilage could be formed by this strategy, the generation of zonal organization in new articular cartilage might be inhibited by the lack of a cartilage-like scaffold for cell accommodation and tissue framework development.

### Single and homogeneous scaffolds

**Fig 3:**
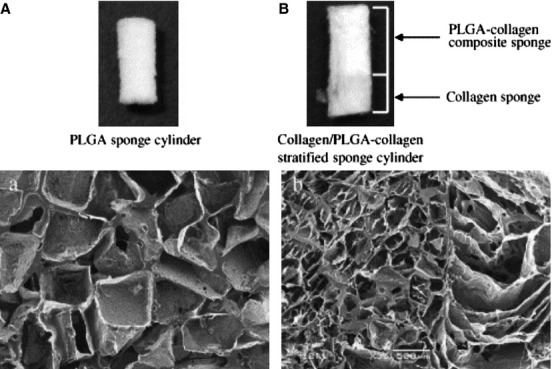
Photos and SEM images of PLGA sponge (A) and collagen/PLGA-collagen bilayered sponge (B) (Reproduced from Chen *et al*. [79] with the permission of Elsevier).

**Fig 4:**
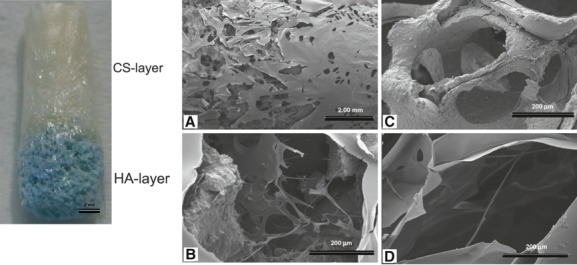
Macroscopic appearance and SEM images of HA/CS bilayered scaffolds: interface (A), typical pore at interface (B), pore of the HA scaffold (C) and pore of the CS layer (D) (Reproduced from Oliveira *et al*. [80], with the permission of Elsevier).

**Fig 5:**
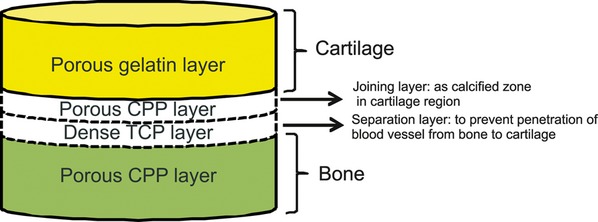
Schematic of the design of the ceramic-gelatin assembly for osteochondral tissue engineering, according to Lien *et al*. [82].

This strategy uses an integrated structure for engineering the complete transition between cartilage and bone layers. These scaffolds incorporate various inclusions or coatings to form unique composite layers in a single structure for simultaneous bone and cartilage regeneration (Fig. 2 (III)). Holland *et al*. [78] studied bilayered scaffolds for osteochondral defects based on degradable oligo(poly(ethylene glycol) fumarate) (OPF) hydrogel. The scaffolds consisted of an OPF layer as bone substrate and OPF/gelatin microparticle loaded TGF-β1 for the cartilage aspect. TGF-β1 was added to initiate the up-regulation of the native chondrocytes activity. It was found that at 14 weeks of implantation in rabbit model, hyaline cartilage with well-organized chondrocytes and intense GAG was apparent. In addition, complete new subchondral bone formation was observed as well as host integration evidence in the OPF bone layer. Accordingly, the quality of repair achieved with these scaffolds confirmed their promise in advancing cartilage repair, encouraging further *in vivo* investigations with these materials. Chen *et al*. [79] prepared bilayered scaffolds for osteochondral tissue engineering, combining biodegradable synthetic polymers and naturally derived polymers. The upper layer of the scaffold was collagen sponge for the cartilage portion; the underlying layer was PLGA/collagen composite sponge for the bone portion. SEM observation of the PLGA sponge indicated a highly porous structure (90.7% porosity) with open pores (Fig. 3A). Moreover, SEM photomicrographs confirmed the stratified structure of the bilayered scaffold (Fig. 3B), one layer was formed by a highly porous collagen sponge and another was a PLGA/collagen composite sponge. Also, it was observed that the collagen sponges in the two layers were connected. The bilayered scaffolds were cultured with canine MSCs, and then a cell/scaffold construct was implanted in an osteochondral defect in canine knees. Histological examination of the implants indicated that cartilage-like and underlying bone-like tissues were regenerated 16 weeks after implantation. The wettability of scaffold surfaces is a very important factor that determines the efficiency of cell seeding in three dimensions. It is generally difficult to deliver cell suspensions in a manner that cells are distributed throughout porous scaffolds made of synthetic polymers because of their hydrophobic surfaces. Therefore, the hydrophilic properties of collagen in the collagen/PLGA-collagen bilayered scaffolds were exploited to facilitate cell seeding and to promote tissue regeneration. Oliveira *et al*. [80] developed novel HA/chitosan (HA/CS) bilayered scaffolds by combining sintering and freeze drying techniques. The scaffolds were cultured with MSCs for osteochondral repair. The interface of the HA/CS bilayered scaffolds was achieved by partially impregnating the porous ceramic layer with the polymer one. As shown in Figure 4A and B, two distinct porous layers were observed and there was good penetration of CS into the HA scaffold, which indicated good bonding between the two layers. The pore structure of the HA layer showed high interconnectivity with pore sizes in the range 50–500 μm (Fig. 4C). In addition, it was possible to observe micro fibres formation in the structure of the CS layer; this could act as an additional anchor-framework to improve cell adhesion (Fig. 4D). The mechanical properties of HA/CS bilayered scaffolds were measured separately. The compressive modulus of the HA structure (153 MPa) indicated its suitability as a bone scaffold and the compressive modulus of the CS structure (2.9 MPa) was in agreement with the value of normal human cartilage (1.9–14.4 MPa). Moreover, *in vitro* cell culture studies demonstrated that both HA and CS layers provided an adequate 3D support for attachment, proliferation and differentiation of MSCs into osteoblasts and chondrocytes, respectively. Gotterbarm *et al*. [81] studied the *in vivo* behaviour of bilayered scaffolds with addition of a growth factor mixture (GFM) in deep osteochondral defects of minipigs. Results were compared with those of untreated defects (without constructs) and with cell-free bilayered scaffolds. The two-layered implant consisted of a porous β-TCP substrate for bone and a fibrous collagen type I/III layer for cartilage. It was shown [81] that treatment with cell-free, bilayered scaffolds improved defect filling and showed more differentiated, reparative tissue at 6 and 12 weeks after implantation. The TCP layer formed cancellous bone within the pores at 6 weeks and the TCP scaffold completely degraded within 52 weeks after implantation. Growth factor treatment improved the mechanical and histomorphological properties of cartilage tissue at 12 weeks after implantation. The highest content of GAG in cartilage defect was found in scaffolds incorporating growth factors in comparison with untreated and cell-free scaffolds [81]. In biomechanical testing, at 6, 12, and 52 weeks after implantation, the highest axial reaction force values after stress-relaxation testing were found after treatment with growth factors, compared with the other treatments. These results reached the highest value at 12 weeks, but decreased after 1 year. Although this study showed the potential benefit of a cell-free, two-layered collagen-TCP construct for the repair of large osteochondral defects, additional deposition of growth factors improved the cartilage repair quality at 12 weeks. However, the outcome was not significantly influenced at 1 year, possibly as a result of degradation and depletion of the growth factors. This result emphasizes the importance and need of longer term studies before final assessment of the suitability of the approach. Lien *et al*. [82] developed a novel osteochondral scaffold based on a ceramic-gelatin assembly for articular cartilage repair and a porous ceramic for bone repair. The novel scaffolds consisted of four layers: a porous ceramic layer as bone component, a dense ceramic layer to prevent blood vessel penetration and to resist shear stresses, a porous ceramic layer to fix bone with cartilage (joining part) and a porous gelatin layer as the cartilage facing component (Fig. 5). This scaffold design was motivated from the problem of achieving improved joining strength at the interface between cartilage and bone. Moreover, chondrocytes must be protected from contact with blood vessels [83], so the penetration of blood vessels from bone to cartilage must be stopped. This will avoid excessive growth of bone and bony spur formation [84], which can impair the function of articular cartilage. The composite scaffolds were seeded with rat chondrocytes and found that cartilage tissue was developed at 4 weeks of culture. GAG and DNA contents increased with culturing time at 1, 2 and 4 weeks. After 4 weeks of culture with chondrocytes, cells had become overgrown and very dense, whereas at 1 week of culture, cells were seen to attach along the surface of pores, but not yet proliferate enough to fill the pores. At 2 weeks of culture, some cells were seen to have undergone cell division. This novel scaffold showed that cartilage tissue could be developed at 4 weeks, and thus this scaffold approach was demonstrated to be feasible for articular cartilage repair. Yunos *et al*. [85] fabricated electrospun PDLLA fibrous coating on 45S5 Bioglass® substrates, to develop a bilayered construct exhibiting rough topography for the improvement of chondrocyte cell attachment. A fibrous surface was tailored by varying the diameter of fibres *via* the adjustment of electrospinning conditions, including polymer concentration, flow rate and operating voltage. An acellular *in vitro* study in simulated body fluid (SBF) showed that HA nanocrystals were formed homogeneously in the fibrous structure after 1 week of immersion, which confirmed the mineralization of PDLLA fibres coated on the surface of 45S5 Bioglass® substrate. This preliminary study was an attractive approach to combine electrospun PDLLA fibres (cartilage side) with PDLLA-coated bioactive glass scaffold (bone side), which formed a stratified scaffold for osteochondral tissue applications [86]. Immersion bilayered PDLLA fibres/PDLLA-coated Bioglass® scaffolds in SBF for up to 4 weeks showed that HA formation was observed in the area of interface, where was the contact between PDLLA mesh and Bioglass® scaffold, increased the strength at the PDLLA fibre/Bioglass® scaffold. Therefore, the possible delamination between two phases was avoided. As mineralized PDLLA fibres, which mimicked the mineralized calcified cartilage, were formed, HA formation was not observed in the PDLLA mesh. This was agreed with a requirement that the cartilage does not mineralize [86]. Moreover, the fibrous topography of PDLLA mesh had a potential to support the attachment, growth and proliferation of a chondrocyte cell line (*e.g*. ATDC5 [86]). It was shown that at day 14 of cell culture, the cells started to migrate through the pores and grow within the 3D network of the PDLLA mesh. These preliminary results indicated that electrospun PDLLA fibres/PDLLA-coated Bioglass® can be a suitable candidate for osteochondral tissue engineering applications. In addition, it had been previously shown that electrospun PDLLA fibrous structures are of general interest in soft tissue engineering applications such as skin regeneration [87]. Cells including fibroblasts, chondrocytes and adult stem cells adhered and proliferated well when cultured on electrospun scaffolds [88, 89]. In case of polylactide derived from lactic acid, the degradation rate of electrospun scaffolds can be tailored by the different kinds of polylactides, according to the ratio of d-, l- and d/l lactide, and their molecular weights [87]. Moreover, their physical and mechanical properties were adjusted for specific tissue applications. Therefore, it was suggested that electrospun polylactide scaffolds are promising candidates for cartilage tissue engineering because of the ability to support chondrogenesis and to provide a balance between degradation rate and mechanical stability [87, 89].

Ohyabu *et al*. [2000] synthesized a novel porous scaffold based on a collagen sponge incorporating HA/chondroitin sulphate composite (pCol-HA/ChS), to form materials which resembled the ECM in bone and cartilage. The HA/ChS composites were prepared by a precipitation method and the suspension of collagen and HA/ChS was freeze-dried to generate porous scaffolds. The combination of ChS with HA made the HA/ChS nanocomposite to have smaller crystals and larger surface area than pure HA. Moreover, pCol-HA/ChS composite scaffolds showed higher porosity (95.5%) and surface area (34.5 m^2^/g) than pCol (86.7% porosity and 6.52 m^2^/g surface area). *In vitro* experiments with composite scaffolds were performed with chondrogenic culture of MSCs. After 2 hrs of seeding, it was found that cell adhesion on the surface and in the interior of pCol-HA/ChS scaffolds was higher than on pCol-HA and pCol substrates because of the rougher surface *i.e*. larger surface area. In addition, MSCs in the pCol-HA/ChS composite scaffolds produced more GAG in cartilage than in pCol-HA and pCol substrates. Therefore, it was demonstrated that ChS included in the porous material influenced the quality of the cartilage matrix. From these results, pCol-HA/ChS composites are expected to be candidates for cartilage scaffolds in place of pCol. Bi *et al*. [91] fabricated biphasic scaffolds for osteochondral tissue engineering exhibiting a stratified structure of collagen–chitosan composite for the chondral phase and collagen–bioactive glass (58S BG) composite for the osseous phase. The stratified scaffolds were connected by cross-linked collagen integrated through pores of two respective phases. After 1 week of seeding MSCs on the scaffold, it was found that cells were able to spread and colonize on the scaffold's surface. Also, the spread cells maintained physical contact with each other. This result showed that this scaffold has good biocompatibility for the seeding and proliferation of MSCs.

In conclusion, single and homogeneous scaffolds are one type of bilayered scaffolds, which are formed in a single composite construct before cell seeding and implantation. Instead of cell-free scaffolds, where neocartilage is generated from seeded chondrocytes on a subchondral support to form cartilaginous layer, single and homogeneous scaffolds are designed to repair osteochondral defects by using tailored bilayered composite structure, which mimic the structure of articular cartilage and subchondral bone tissue. The integration at the interface between engineered cartilage and the subchondral bone part is developed *via* fabrication methods to form the physical integration before cell seeding and implantation [81, 90].

### Single but heterogeneous scaffolds

**Fig 6:**
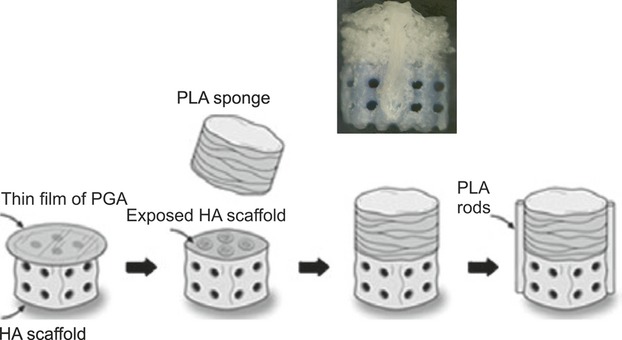
Bilayered composite scaffolds produced by Schek *et al*. [93]. Poly-l-lactic acid (PLLA) rods were used to join the polymer and ceramic phases of the composite. One face of the ceramic was coated with a thin film of poly-gamma-glutamic acid (PGA) (A). The film was removed from the circumference and 10 μl of PLA (7.5% in methylene chloride) was applied (B). The polymer sponge was pressed onto the ceramic scaffold, allowing the solubilized PLLA to serve as adhesive (C). PLLA (25% in methylene chloride) struts were extruded on two opposite sides of the scaffold to further stabilize the composite (D) (Reproduced from Schek *et al*. [93] with the permission of John Wiley and Sons).

This category of bilayered scaffolds is composed of two distinct but integrated layers for the cartilage and bone regions [2] (Fig. 2 (IV)), as opposed to generating composite osteochondral grafts by combining independent cartilaginous and bone-like components. Following this approach, Aydin *et al*. [90] produced osteochondral scaffolds based on PGA woven felt for the cartilage part, collagen and HA-coated porous PLLA/PCL foam for the bone part and PLLA/PCL layer as the cartilage–bone interface. The pore structure of the bone scaffold was developed by additional generation of vertical channels to deliver stem cells and blood from bone marrow when implanted. *In vitro* culture of L929 mouse-fibroblast cells into osteochondral scaffolds showed high value of cell viability and low degree of cytotoxicity in both cartilage and bone scaffolds. However, cell behaviours and tissue regeneration were not shown in this study. The interface of scaffolds was not carefully analysed in the *in vitro* study. Sherwood *et al*. [52] developed an innovative osteochondral composite scaffold consisting of porous D,L-PLGA/L-PLA in the form of macroscopic staggered channels seeded with ovine articular chondrocytes for the cartilage side and porous L-PLGA/TCP for the bone layers. The staggered channels facilitated the seeding of chondrocytes into the centre of the cartilage portion, allowed transport of nutrients to the cells and removal of cellular and polymer degradation by-products. These composite scaffolds promoted cell attachment and the formation of cartilage was observed after 6 weeks of *in vitro* culture. Cartilage and bone parts were connected by a gradient of materials and pores, which formed a transition region to prevent delamination after transplantation. The bone region had compressive strength (2.5–13.5 MPa) close to values of cancellous human bone, indicating that the bone part had desirable mechanical properties for *in vivo* application. Cao *et al*. [55] attempted to prepare osteochondral scaffolds by designing three dimensional load-bearing structures. 3D porous PCL scaffolds were co-cultured with osteoblasts and chondrocytes. They found that both cell types proliferated, migrated, linked in their compartment, integrated at the interface, and produced different ECM under co-culture conditions. However, the final actual phenotype of the osteoblasts and chondrocytes in the PCL scaffolds was not observed, for example, by DNA sequencing experiments. Schek *et al*. [93] produced bilayered composite scaffolds with suitable biological and mechanical properties by using image-based design (IBD) and solid free-form (SFF) fabrication. The combination of these techniques created scaffolds that were load-bearing and matched defect site geometry. The two phases of the scaffolds, composed of PLLA and HA, were assembled prior to cell seeding and implantation. These composite scaffolds were stabilized by using two bonded cylinders of PLLA (Fig. 6D), and a thin PGA film was deposited between the two layers to prevent cell migration (Fig. 6A). The bilayered scaffolds were seeded with osteogenic cells in the ceramic phase and chondrocytes in the cartilaginous phase. Scaffolds were then immediately implanted in mice. Following implantation, the composite scaffolds were seen to promote the growth of bone and cartilage, and a mineralized interface tissue was formed. The PLLA rods connecting the two phases were in intimate contact with both the PLLA and HA phases and the composite scaffolds could withstand surgical implantation. The presence of GAG in the PLLA sponge phase was observed by staining, indicating chondrocyte synthesis of cartilage matrix. Small pockets of cartilage were also observed invading the pore of the ceramic phase. Within the pores of the HA phase, bone was observed together with other tissues such as fibrous tissue and fat. These results therefore demonstrated tissue formation with different cell seeding and the development of the bone–cartilage interface on bilayered composite scaffolds [93]. Ghosh *et al*. [94] studied porous bilayered scaffolds for osteochondral tissue engineering made of PLLA/starch blends for the cartilage part and PLLA/HA or Bioglass® for the bone part. This work considered that starch might provide capability of water uptake and HA/Bioglass® should enhance bioactivity and HA formation on the bone side. The interface between cartilage and subchondral bone was integrated by a melt-based process. Moreover, it was suggested that the use of PLLA in both sides was to increase the bonding between them. The use of compression moulding followed by particle leaching was proposed to generate porous scaffolds with controllable porosities. For the cartilage region, a blend of PLLA and starch exhibited adequate hydration capability and for the bone region, PLLA reinforced with HA/Bioglass® showed the required stiffness and strength. The presence of HA/Bioglass® also induced formation of a CaP layer *in vitro,* as expected. However, further cell culture and *in vivo* studies are necessary to demonstrate that the developed constructs could have potential to be used in regeneration of osteochondral defects in realistic patient conditions. Jiang *et al*. [85] improved a stratified osteochondral scaffold by focusing on the regeneration of a calcified interface. The bilayered scaffolds based on chondrocytes-containing agarose hydrogel for cartilage and osteoblasts-containing composite microspheres of PLGA and 45S5 Bioglass® for bone were fabricated in a cylindrical mould, and the interface was formed by chondrocytes embedded within a hybrid phase of gel and microspheres. An *in vitro* study showed that at the integrated area, PLGA/Bioglass® composite promoted chondrocyte mineralization and led to the formation of a calcified interface. The construct regions were well integrated with each other and were maintained *in vitro* without delamination over time. Moreover, the chondrocytes-loaded agarose hydrogel promoted the formation of cartilage-like ECM and the PLGA/Bioglass® composite microspheres supported collagen deposition by osteoblasts. However, the encapsulation of chondrocytes in agarose hydrogel for cartilage to improve the mechanical properties of the construct was not successfully achieved as the highest Young's modulus achieved was about 20 kPa, which is still lower than that of native cartilage (0.2–0.3 GPa) [96]. To further improve the mechanical strength, future studies were suggested to focus on utilizing biochemical factors and mechanical stimuli. Kinikoglu *et al*. [97] fabricated fibre-foam bilayered scaffolds based on elastin-like recombinamers (ELRs)/collagen fibrous mesh and collagen porous foam. ELRs/collagen fibres were spun directly on collagen foam, which was fabricated by freeze drying technique, leading to the effective connection between the fibre mesh and the surface of foams thus forming a bilayered structure. Moreover, culture of fibroblasts in ELRs/collagen fibrous mesh showed cell proliferation because of ELRs, which were enriched with short peptides having bioactivity and also improved cell attachment.

In conclusion, single but heterogeneous scaffolds involve seeding of different cells specifically for cartilage and bone tissues. Likewise, single and homogeneous scaffolds, heterogeneous scaffolds are formed before cell-seeding and implantation. The interface between the cartilage and bone parts is developed during fabrication and laminated biological integration occurs by cell growth and cell distribution in the porous structure. In both homogeneous and heterogeneous scaffolds, the scaffold architecture can be tailored for cell adhesion and tissue organization, for example by, functionally graded scaffolds with gradients in pore size, porosity and material composition to achieve the required properties in cartilage and bone tissues. Despite the variety of materials, scaffold designs and cells that have been investigated for osteochondral tissue engineering, an optimal strategy has not yet emerged. Therefore, more research efforts are needed to find suitable combinations of materials and methodologies that can be transferred to clinical practice.

## Discussion

Many biomaterial-based approaches are being put forward which attempt to create bilayered scaffolds suitable for regeneration of osteochondral defects. The important purpose is to design scaffolds that mimic the tissue of both components of the joint: cartilage and subchondral bone. Bioactive ceramics and glasses are considered optimal candidates for the bone component because of their mechanical rigidity as well as high bone bonding ability. The polymeric phase confers toughness and plasticity, and it is suitable as substrate for providing the contact with cartilage tissue. Therefore, the combination of bioceramic and polymeric phases generates suitable composite materials with adequate biological and mechanical properties attractive for osteochondral tissue engineering. There is evidence that the polymeric or bioceramic phases taken individually could not reach the optimal scaffold structure and functionality required for osteochondral tissue engineering. For example, Chen *et al*. [79] prepared a collagen/PLGA-collagen stratified sponge cylinder composite to improve the surface wettability and cell seeding efficiency because biodegradable synthetic polymers have hydrophobic surfaces, which hinder cell seeding and penetration. Cartilage-like and underlying bone-like tissues were well integrated with surrounding tissue and in the PLGA-collagen composite sponge portion, PLGA sponge served as the skeleton, providing the composite with sufficient mechanical strength while collagen formed within the pores of the PLGA sponge promoted cell adhesion and proliferation. It was concluded [79], however, that it is difficult to deliver cells into porous scaffolds made of biodegradable polymers such as PLA and PLGA because of their hydrophobic surfaces. Thus, the combination of bioactive inorganic materials such as HA, TCP and bioactive glasses with a polymer matrix forming composite materials should overcome this problem [98]. Designed composites incorporating bioactive glasses/ceramics and biodegradable synthetic polymers seem to be well suited to generate scaffolds for osteochondral tissue engineering. Ceramics and polymers that degrade *in vivo* should be chosen for designing biocomposite scaffolds because, while the release of acidic degradation products from certain polymers can cause inflammatory reactions, the basic character of the degradation of the inorganic materials would buffer the acidic products and should help avoid the formation of an unfavourable environment for cells as a result of a much decrease in pH [99]. Bioactive glasses are being highly considered for bone tissue engineering scaffolds because they are highly surface reactive and can rapidly produce HA surface layers that bond to bone *in vivo* [100]. Bioactive glass of composition 45S5 Bioglass® (in wt%: 45% SiO_2_, 24.5% Na_2_O, 24.4% CaO, and 6% P_2_O_5_) exposes Ca, Si, Na and P ions, which have been shown to activate genes in osteoblast cells, thus stimulating new bone formation *in vivo* [99, 101]. In addition, bioactive glasses in porous form are biodegradable, highly osteoconductive and suitable for cell delivery. However, Bioglass® is mechanically weak and has low fracture resistance, which causes Bioglass®-based scaffolds to be inadequate for load bearing applications [20]. To minimize their shortcomings, Bioglass® can be combined with biodegradable polymers, such as PLA, PGA and PLGA, forming biodegradable polymer/Bioglass®-based composite scaffolds [48]. Polymer coatings improve the mechanical properties of highly porous inorganic scaffolds, *e.g*. they lead to increased work of fracture [45, 48, 75, 102]. The addition of polymers might have extra functions in the scaffold. For example, biodegradable polymers can act as carrier for biomolecules and growth factors hence increasing the performance of tissue engineering constructs. Chen *et al*. [45] developed 45S5 Bioglass®-based scaffolds with improved fracture resistance by coating the scaffolds with PDLLA. The compressive strength of PDLLA-coated 45S5 Bioglass®-based scaffolds was slightly higher compared with uncoated scaffolds, but the work of fracture of coated foams was significantly improved. Moreover, the bioactivity of Bioglass®-based scaffolds was maintained after coating. Bretcanu *et al*. [103] studied the biocompatibility of 45S5 Bioglass®-based scaffolds coated with poly(3-hydroxybutyrate) (P(3HB)). It was shown that these scaffolds were highly bioactive, as, after immersion in SBF for a few days, HA was formed on the surface. It was also observed that P(3HB)-coated scaffolds exhibited higher compressive strength than uncoated scaffolds. Moreover, the experiments confirmed that relevant cells were able to proliferate and grow on the scaffolds. Thus, there is evidence that the polymeric phase in polymer-coated bioceramic scaffolds is useful to overcome the brittleness problem of bioceramic-based scaffolds, considering that the polymeric phase in the form of thin layers or filaments can bridge cracks formed in the bioceramic phases [45, 99]. A major drawback of several scaffold approaches for osteochondral tissue engineering is that they fail to produce a functional interface between bone and cartilage. Therefore, it has been suggested that besides designing osteochondral scaffolds and mimicking the 3D ECM, mimicking the characteristics of the interface between bone and cartilage is the key challenge, given the complex nano- and macro-architecture of the ECM [103]. Electrospinning is a promising method for producing artificial ECM tissues for supporting cells such as MSCs. This technique is technically feasible for the fabrication of filaments ranging from the nanometre to micrometre scale, and comprehensive reviews are available [89, 104–108]. Synthetic polymers such as PLA (Janjanin *et al*. [109], Ma *et al*. [110], Jeong *et al*. [111]), PCL (Yoshimoto *et al*. [112], Shin *et al*. [113], Li *et al*. [114], Neves *et al*. [115]) and PLGA (*Li et al*. [116], Mouthuy *et al*. [117]), and natural polymers such as collagen (Song *et al*. [118]), fibrinogen and poly(hydroxyalkanoates) (PHB, PHBV, P4HB, PHBHHx, PHO) (Bretcanu *et al*. [103]), have been processed by electrospinning into fibrous non-woven scaffolds to support cell growth and differentiation [119, 120]. Srouji *et al*. [121] prepared 3D nanofibrous multilayered scaffolds to mimic natural ECM by using electrospinning of PCL and bovine collagen type I for MSCs support. It was found that the novel 3D nanofibre multilayered constructs are able to contain efficient cell mass necessary for successful *in vivo* grafting. Jeong *et al*. [111] produced nanofibrous PLA/nano-HA composite scaffolds by using electrospinning technique for guided bone tissue regeneration. The polymer coating on bioceramic composites limits the exposure of the bioceramic on the surface of the scaffold, decreasing the chance that cells make contact with the bioactive component. Nanosized features of electrospun scaffolds have demonstrated an assembly of non-woven macro/nanofibres mimicking the structure of natural ECM. The incorporation of HA nanoparticles showed the formation of smooth nanofibres with high pore volume and interconnecting pores. An *in vitro* cell culture study, in which pre-osteoblasts (MC3T3-E1) were used as a model cell type, showed that cells maintained viability and proliferated continuously up to 3 weeks, indicating that PLA/HA nanofibres are effective scaffolds for the growth of pre-osteoblasts. Moreover, these composite scaffolds showed promise as a temporary substrate for osteoblast culture in guided regeneration of bone tissue. Electrospun PLLA/nano-HA composite fibrous scaffolds for bone tissue engineering were also studied by Chuenjitkuntaworn *et al*. [122]. Their potential as substrates for bone cell culture was assessed. The porosity of these fibrous scaffolds was high (97–98%). *In vitro* studies were carried out in which PLLA/HA fibrous scaffolds were cultured with MC3T3-E1 mouse pre-osteoblastic cells. The results showed that the presence of HA in PLLA fibrous scaffolds not only promoted attachment and proliferation of pre-osteoblastic cells but also increased the extent of mineralization after 3 weeks culture. Song *et al*. [118] studied collagen-apatite nanocomposite scaffolds for bone regeneration by using electrospinning technique. They concluded that electrospun fibrous layers of collagen-apatite nanocomposite might be useful as a cell supporting substrate for bone regeneration, but the materials were not studied for cartilage repair. A study of 3D nanofibrous scaffolds for cartilage tissue was presented by Li *et al*. [114]. They fabricated a nanofibrous scaffold made of PCL and examined the ability of the scaffold to support chondrogenesis of MSCs *in vitro* in the presence of TGF-β. The morphological analysis showed that 3D MSC-seeded constructs displayed a cartilage-like morphology, containing chondrocyte-like cells surrounded by cartilaginous matrix. Quantitative biochemical assays also confirmed the continuous synthesis of GAG during the 3 weeks of culture time. Because biodegradable nanofibrous scaffolds have a high surface to volume ratio, they provide a favourable 3D porous space to accommodate cells at high density. It was demonstrated that MSCs seeded and cultured in nanofibrous PCL scaffolds successfully undergo chondrogenesis *in vitro* [114, 123]. It was proposed therefore that 3D nanofibrous PCL scaffolds are suitable candidate bioactive carriers for MSCs transplantation in tissue engineering-based cartilage repair. Thorvaldsson *et al*. [119] studied the electrospinning technique to produce porous scaffolds for cartilage regeneration. The electrospinning technique was used to coat single microfibres with nanofibres. Nanofibrous PLA-coated microfibrous PCL scaffolds exhibited the combined benefits of tailored porosity (95–97%) for cellular infiltration and nanostructured surface morphology for cell growth. Moreover, the 3D structure of electrospun scaffolds allowed cells to fully differentiate, leading to maintenance of normal cell activity that is not possible in 2D environments. These nanofibre-coated microfibre scaffolds with pore sizes of ∼100 μm showed a significant improvement in cellular infiltration. Janjanin *et al*. [109] evaluated the potential of using MSCs seeded into electrospun PLLA scaffolds, which were then cultured in a bioreactor to engineer cartilage. Chondrogenesis was induced with TGF-β1 or IGF-I. The constructs showed formation of hyaline cartilage, tissue integrity and shape retention after 42 days' culture. Cartilage matrix gene expression and amount of GAG were seen after 42 days' culture and increased with time. Therefore, these nanofibrous scaffolds were confirmed to be applicable as a highly functional biomaterial matrix supporting cell distribution, efficient cell differentiation and ECM expression. In a related investigation, Mouthuy *et al*. [117] investigated the potential of the electrospinning technique to prepare 3D PLGA/collagen-containing nano-HA constructs, mimicking the zonal organization of the bone–cartilage interface. It was shown that fibre membranes produced by electrospinning could mimic the ECM and they could be used as a support for tissue culture. Moreover, electrospun membranes could be tailored in terms of architecture and composition to optimize cell response. The presence of collagen and HA increased the biocompatibility of scaffolds, also enhancing their stiffness and hydrophilicity. Chitosan/PCL blend fibre mesh scaffolds were studied for cartilage repair by Neves *et al*. [115]. Microfibres were obtained by wet-spinning and then these fibres were folded into cylindrical moulds and underwent thermal treatment to obtain the scaffolds. The PCL component led to a higher fibre surface roughness and increased compressive strength of the scaffolds. In terms of biological behaviour, after culturing bovine articular chondrocytes, cartilaginous ECM formation was observed and the scaffolds supported neocartilage formation, as demonstrated by an increase in GAG production. Overall, electrospinning is a promising technique that produces polymeric fibrous structures comparable to the ECM of many tissues. Interestingly, nanofibrous scaffolds adsorb more selective proteins than solid walled scaffolds, indicating enhancement of cell adhesion [124]. Additionally, electrospinning is a simple and versatile processing method that enables the control of fibre size and orientation by varying polymer concentration, solvent optimization and processing parameters. However, fabricating 3D shapes and controlling porosity and pore shape are remaining challenges that have limited the further use of electrospinning in tissue engineering applications [125–127]. To fabricate 3D fibrous scaffolds, a collector plate can be replaced with a rotating cylinder [127] or a 3D columnar collector [128], or the fibrous meshes can be rolled up to produce a tubular scaffold [126]. It is also well known that the small pore size produced by electrospinning limits cellular infiltration and tissue ingrowth into fibrous scaffolds. Diverse techniques are being developed to overcome this problem such as variation of electrospinning parameters, use of rotating collector and application of wet electrospinning, as reviewed by Kovacina *et al*. [129] and Teo *et al*. [130]. For example, the porosity of electrospun meshes in 3D can be increased by using a salt leaching method [126]. In addition, vapour or heat treatment [126] can be applied to improve the mechanical properties of electrospun fibrous meshes. Indeed, technological advances in electrospinning are expected to enhance its applications in tissue engineering scaffold production. Besides the discussed electrospinning process, fibres can be generated also by alternative techniques. For example, Steck *et al*. [131] and Reiband *et al*. [132] produced scaffolds by using flock technology, which is based on electrostatic application of fibres fed onto various material surfaces that are covered by an adhesive [132]. Flocking applies short fibres (0.3–5 mm in length) to a substrate, the fibres being aligned in an electrostatic field and accelerated towards the adhesive-covered substrate [132]. Reaching the adhesive, the fibres are fixed in perpendicular direction to the substrate providing a uniform flock coating [133]. In the study of Steck *et al*. [131], anisotropic scaffolds for cartilage repair composed of collagen type I as substrate, gelatin as adhesive and parallel-oriented polyamide fibres arranged vertically to the substrate supported a cartilaginous phenotype *in vitro*. The results showed that MSCs adhered and proliferated well on the scaffolds and cell vitality remained high over time. Proteoglycans and collagen type II were observed in the flock scaffolds seeded with chondrocytes, indicating that these scaffolds could be appropriate candidates for cartilage repair. Moreover, in terms of mechanical properties, flock scaffolds exhibited higher initial hardness than a clinically applied collagen scaffold, which increased further in MSC-loaded flock composites during chondrogenesis. However, these scaffolds have not been studied *in vivo* yet. While the cartilage component of osteochondral constructs is designed as a blood vessel-free tissue substitute analogue to native avascular cartilage, an additional challenge for engineering the bone part has to be addressed in the future: vascularization strategies for *in vivo* integration of the engineered bone part. Although osteointegration of smaller constructs might be successful, initial perfusion of central parts within larger bone constructs after transplantation may be insufficient to provide cell survival. Thus, pre-vascularization may be deemed necessary when it comes to *in vivo* application of larger osteochondral bone constructs or when such a construct is implanted in a recipient site with reduced blood flow (following irradiation or chronic infection, *e.g*.). Different approaches to vascularization in the field of bone tissue engineering have been pursued in the past. Tanaka *et al*. [133] established a microsurgical animal model, the rat “AV-loop model”, as a promising approach for tissue engineering purposes, facilitating the generation of axially vascularized tissue. In contrast to randomly vascularized tissue constructs, axially vascularized constructs can be transplanted with re-connection of their central vascular axis to blood vessels at the recipient site, thus providing sufficient blood supply even to central parts of the tissue engineered construct immediately after its transplantation. In the subsequent years this strategy has evolved in the field of bone tissue engineering with application of different bone substitutes in the rat AV-loop [134] as well as transplantation of osteoblastic cells [135] and administration of angiogenic growth factors [136]. To transfer tissue engineering developments from bench to bedside, scaling up of established small animals models and developing new large animal models are the next consequent steps towards future clinical application. Cheng *et al*. [137], for example, described the generation of a vascularized bone construct *in vivo* by implanting an isolation chamber filled with bone substitute directly over the cambium layer of the rib periosteum in a large animal sheep model. The small animal rat AV-loop model has also been translated to large animal models: axial vascularization of different bone substitutes as tissue engineering matrices was demonstrated in a newly developed sheep AV-loop model [138, 139]. Eweida *et al*. [140] proposed a different model for generation of axially vascularized bone constructs: an AV-loop model in the goat, based on its facial vessels. While successful axial vascularization of bone constructs was achieved in these different models, generation and transplantation of functional bone tissue with appropriate biomechanical properties still have to be shown. In this context, vascularization strategies developed for pure bone tissue engineering may also play a crucial role for future *in vivo* application of large osteochondral tissue constructs.

## Conclusions

By describing the wide variety of approaches being investigated for osteochondral tissue engineering, numerous challenges are highlighted, which are associated with defect site, physicochemical/mechanical properties of materials, cell types and interaction between them, as well as interaction between scaffolds and native tissue. These challenges are also influenced by the complexity of interface tissue composition and properties because of the required integration of cartilage and underlying subchondral bone, which exhibit significantly different biomechanical structure and functional properties. Based on the particular structure to be regenerated in this branch of interface tissue engineering, bilayered composite scaffolds are being developed with the aim to facilitate the temporary mimicking of the local tissue organization at the interface between both tissues. The pore structure of scaffolds is an important factor for cell differentiation and proliferation, and for regeneration of new tissue. The optimized size of pore interconnection of cartilaginous scaffolds should be less than that of subchondral bone scaffolds because articular cartilage is normally fed by articular fluid, whereas bone is fed by the nutrients from blood circulation [73]. The porous structure of scaffolds can be tailored *via* carefully developed material fabrication methods. Moreover, an appropriate biomaterial for cartilage repair must be compressible and flexible to avoid damage of host cartilage in contact with the engineered scaffold. However, engineered scaffolds must have enough mechanical strength to maintain long-term stability. Another important factor to be considered is to develop cartilage–bone interfaces that do not delaminate after implantation. Strong cartilage–bone interfaces can be generated by promoting biological and physical integration, both *in vitro* and *in vivo*. In terms of biological integration, many approaches have shown that cartilage and bone layers can be connected by new formed ECM from chondrocyte-seeded scaffolds. In addition, it is suggested that the formation of zonal organization in neocartilage is necessary for a successful integration of the interface, following the fact that mineralized calcified layer is the interface, which connects hyaline articular cartilage and subchondral bone. The local formation of neocartilage depends on the interaction between chondrocytes and the engineered cartilaginous component, which depends on surface chemistry and topography of the scaffold, as well as on pore size and porous structure, and on the degradation rate of the biomaterials used. In terms of physical integration, the interfacial shear strength of bilayered scaffolds can be improved by the binding interaction between the respective scaffolds for cartilage and bone tissues before cell seeding and implantation. In some studies, scaffolds for cartilage and bone are connected by using additional glues such as fibrin glue and polymer thin films to stabilize the bilayered structures for *in vitro* and *in vivo* studies. Novel strategies suggest the possibility to connect distinct layers forming a connected stratified scaffold by using polymer–polymer bonding, infiltration of polymer into porous bioceramic scaffold, and simultaneous adjustment of fabrication techniques. However, the ideal bilayered scaffold for osteochondral tissue engineering has not been developed yet. The clinical relevance in this area is in progress as well. Indeed, osteochondral tissue engineering is at the very interface of several research areas including materials science, cell and molecular biology and clinical medicine. According to the basic components discussed, besides cell seeding on scaffolds for chondrogenic and osteogenic potential, the incorporation of specific growth factors is a promising option to regulate cell differentiation and also to improve the mechanical properties of the cartilage repair tissue after implantation. Selection of specific cell types for seeding into scaffolds depends on the application and material structure. Osteochondral repair involves significant challenges as osteochondral constructs must avoid the risk of cell phenotypic changes and the regeneration of neo-cartilage tissue must achieve the structural organization of the native tissue. In this context, the consideration of *in vivo* studies in appropriate small and large animal models with variation in defect thickness is needed to investigate integration ability of scaffolds, vascularization of the bone part, possible inflammatory response and long-term stability to translate *in vitro* data into clinically relevant approaches.
